# Molecular and clinical characterization of hypervirulent *Klebsiella pneumoniae* isolates from individuals with urinary tract infections

**DOI:** 10.3389/fcimb.2022.925440

**Published:** 2022-08-08

**Authors:** Jun Li, Mengli Tang, Zhaojun Liu, Fengjun Xia, Changhang Min, Yongmei Hu, Haichen Wang, Mingxiang Zou

**Affiliations:** ^1^ Department of Clinical Laboratory, Xiangya Hospital, Central South University, Changsha, China; ^2^ National Clinical Research Center for Geriatric Disorders, Xiangya Hospital, Central South University, Changsha, China

**Keywords:** hypervirulent *K. pneumoniae*, antibiotic resistance, urinary tract infections, clinical characterization, whole-genome sequencing

## Abstract

Despite being a significant public health concern, hypervirulent *Klebsiella pneumoniae* (hvKP) has rarely been investigated in urinary tract infections (UTIs). To investigate the molecular and clinical characterization of hvKP in UTIs, we collected *K. pneumoniae* strains and clinical data from patients with UTIs. HvKP was confirmed by virulence-related genes and the *Galleria mellonella* model and sequenced by next-generation sequencing. Our data showed that 30/121 isolates were hvKP [17 carbapenem-resistant hypervirulent *K. pneumoniae* (CR-hvKP), 12 hvKP, and 1 extended-spectrum β-lactamase-producing hvKP]; these had higher resistance to most antimicrobials and were more likely to cause complicated UTIs (cUTIs). Notably, the mucoid phenotype-regulating genes *_p_rmpA* and *_p_rmpA2* were truncated in 3 and 19 hvKP, respectively. Eight serotypes were detected and divided into three groups: K64 (*n* = 17), K1/K2 (*n* = 6), and others (*n* = 7). Furthermore, 16/17 K64 hvKP isolates were CR-hvKP but with a lower mortality rate of *G. mellonella* as the truncated *_p_rmpA/_p_rmpA2* incurred high fitness cost to the isolates. In addition, all K64 isolates belonged to ST11 with the same cluster, and in two of these strains (KP88 and KP92) *bla*_KPC-2_ gene was successfully transferred to EC600. Genetic environment analysis showed that IS*26*–*tnpR*–IS*Kpn27*–*bla*_KPC−2_–IS*Kpn6* may be the core structure in the horizontal transfer of *bla*_KPC-2_. The highest mortality rate among the infected *G. mellonella* was observed in the K1/K2 group. In conclusion, hvKP had a higher resistance rate and was more likely to lead to cUTIs. Convergence of hypervirulence and carbapenem resistance in a transmissible ST11 clone of K64 *K. pneumoniae* was mediated by a plasmid in UTIs. Therefore, surveillance of hvKP in UTIs should be strengthened.

## Introduction

Globally, more than 150 million people are affected with urinary tract infections (UTIs) yearly, making it as the most frequent infections (Öztürk and Murt, 2020). To confirm a diagnosis of UTI, a combination of positive urine culture, pyuria, and the relevant signs and symptoms is needed. A characteristic finding of UTIs is the presence of microbial pathogens in the urinary tract; it is divided into symptomatic UTI (with associated signs and symptoms) and asymptomatic bacteriuria (without associated signs and symptoms) (Mody and Juthani-Mehta, 2014). Typically, symptomatic UTI is divided into complicated and uncomplicated UTIs, and in complicated UTIs (cUTIs), patients often have functional or structural abnormalities or systemic diseases (e.g., renal insufficiency, immunodeficiency, or diabetes); furthermore, they are at a greater risk of developing systemic infection symptoms, such as fever and sepsis (Mody and Juthani-Mehta, 2014).

Previous studies revealed the following as some of the pathogens causing UTIs: *Escherichia coli*, *Klebsiella pneumoniae*, *Enterococcus faecium*, and *Enterococcus faecalis* (Nicolle, 2015). *K. pneumoniae* was the second most prominent pathogen. Hypervirulent *K. pneumoniae* (hvKP) is a more easily transmitted, more pathogenic, and a more fatal variant of *K. pneumoniae* (KP) (Lee et al., 2017; Russo and Marr, 2019). Notably, hvKP can cause infection at multiple locations: endophthalmitis, abdominal disease, thoracic disease, central nervous system disease, and genitourinary tract infections (Russo and Marr, 2019). In recent times, its multidrug-resistant and extensively drug-resistant isolates have also been detected in UTIs (Mataseje et al., 2019; Zafar et al., 2019; Zhu et al., 2020). The finding that hvKP infection is more likely to cause serious consequences and bacteremia than classical *K. pneumoniae* (cKP) as a causative agent of UTIs is particularly concerning (Kim et al., 2017).

Although hvKP has been reported in UTIs, systematic reports of hvKP in UTIs have been rare (Zafar et al., 2019; Taraghian et al., 2020). Therefore, herein, we obtained hvKP isolates from patients with UTIs and compared the clinical and molecular characteristics of these isolates with those of cKP to provide laboratory basis for the empirical treatment of UTIs.

## Methods

### Materials and methods

We collected urine samples from patients with symptomatic UTIs from October 2019 to October 2020 at Central South University’s Xiangya Hospital located at Changsha, China. Patients were considered as having symptomatic UTI if they exhibited typical signs and symptoms of a UTI, with bacteriuria [≥10^5^ colony forming units (CFUs)/mL] and pyuria (≥10 white blood cells/high-powered field) (Mody and Juthani-Mehta, 2014). Furthermore, we collected demographic data as well as information pertaining to underlying diseases and clinical manifestations. Freshly voided midstream urine (5–10 mL) was aseptically collected in wide-mouthed, sterile plastic bottles having a tight cap. Then, 0.001 mL of uncentrifuged, uniformly mixed samples were aseptically inoculated onto blood agar plates (Guangzhou Boret Biotechnology Co., Ltd., Guangzhou, China) using a calibrated inoculating loop. After incubating overnight for 24–48 h at 37°C, any significant growth was checked by counting colonies. Bacteriuria was indicated by the presence of colonies yielding a bacterial growth of 10^5^ CFUs per one milliliter of urine (Mody and Juthani-Mehta, 2014). Matrix-assisted laser desorption/ionization–time-of-flight mass spectrometry (MALDI–TOF MS; Bruker Daltonics GmbH, Bremen, Germany) was used to identify all isolates, and the quality control strain used was *E. coli* ATCC 25922 (National Center for Clinical Laboratories, Beijing, China).

HvKP was screened as carriage of at least two of the *_p_rmpA*, *_p_rmpA2*, *iroB*, *peg344*, and *iucA*, and confirmed with *G. mellonella* infection model (Liu et al., 2020).

### Antimicrobial susceptibility testing

The minimum inhibitory concentrations (MICs) of the antimicrobial agents used were determined using the broth microdilution test according to the manufacturer instructions. The agents included cefepime (FEP), ceftazidime (CAZ), levofloxacin (LEV), ceftriaxone (CRO), piperacillin/tazobactam (TZP), meropenem (MEM), amikacin (AK), imipenem (IPM), ciprofloxacin (CIP), gentamicin (GEN), aztreonam (ATM), trimethoprim/sulfamethoxazole (SXT), nitrofurantoin (F), ceftazidime/avibactam (CZA), tigecycline (TGC), and polymyxin B (PB), and all of these agents were obtained from Hangzhou Kangtai Biotechnology Co., Ltd.. The guidelines of the Clinical and Laboratory Standards Institute’s (2020) were used to interpret susceptibility breakpoints (CLSI, 2020). The breakpoints indicated by the US Food and Drug Administration determined the MIC of TGC. *E. coli* ATCC 25922 was used as the quality control strain.

### Phenotypic screening of extended-spectrum β-lactamase

The presence of ESBLs was evaluated using CLSI criteria for screening these bacteria using the double-disk diffusion method. An increase of >5 mm in the inhibition zone of the ceftazidime–clavulanic acid disk when compared with the ceftazidime alone disk was interpreted as phenotypic evidence of ESBL production.

### Detection of hypermucoviscosity and virulence-associated genes

The hypermucoviscosity of *K. pneumoniae* was determined by string test as described previously (Li et al., 2020). Briefly, after subculturing *K. pneumoniae* isolates overnight at 37°C on 5% sheep blood agar, a bacterial loop was used to stretch a “string” of mucous from the bacterial colony. A mucoviscous string longer than 5 mm indicated hypermucoviscosity (Li et al., 2020).

For all *K. pneumoniae* strains, their genomic DNA was extracted using overnight-cultured strains using the boiling method (Li et al., 2016). For detecting capsular serotype genes (K1, K2, K5, K20, K54, K57, and K64), *_p_rmpA*, *_p_rmpA2*, *iroB*, *peg344*, and *iucA*, we performed by polymerase chain reaction (PCR) as described previously (Yu et al., 2018; Liu et al., 2020). This was followed by subjecting the positive PCR products to direct Sanger sequencing.

### Galleria mellonella infection model

We used the *G. mellonella* infection model (Tianjin Huiyude Biotech Company, Tianjin, China) for evaluating the virulence of the collected isolates (Zhou et al., 2018). After adjusting the *K. pneumoniae* overnight cultures to 1 × 10^8^ CFUs/mL with the use of phosphate-buffered saline, the bacterial culture (10 μL) was injected into *G. mellonella* larvae, followed by incubation of the larvae in the dark for 5 days at 37°C; the survival was monitored during this period. *K. pneumoniae* ATCC 700603 and *K. pneumoniae* NTUH-K2044 were the low- and high-virulence control strains used, respectively. All experiments were performed in triplicate.

### Biofilm formation assay

To test if KP isolates formed a biofilm, the previously described crystal violet staining method was implemented (Coffey and Anderson, 2014). The absorbance was observed at 570 nm, and for each assay performed in triplicate, data are represented in the form of mean ± standard deviation (SD). *K. pneumoniae* NTUH-K2044 and ATCC 700606 were the positive and negative controls used, respectively. The optical density (OD) cut-off (ODc) value was calculated according to the parameters defined by Ragupathi et al. (Devanga Ragupathi et al., 2020) as follows: ODc = average OD of the negative control + (3 × SD of the negative control). Using the OD results, the biofilm formation ability was categorized as follows: (1) medium biofilm producer (4 × ODc ≥ OD > 2 × ODc); (2) strong biofilm producer (OD > 4 × ODc); (3) non-biofilm producer (OD ≤ ODc); and (4) weak biofilm producer (2 × ODc ≥ OD > ODc).

### Whole-genome sequencing (WGS)

Genomic DNA was isolated using MagAttract HMW DNA Kit (Qiagen, Hilden, Germany) and subjected to high-throughput next-generation sequencing (NGS) on a HiSeq 2000™ platform (Illumina Inc., San Diego, CA, USA) with 2 × 100-bp paired-end reads and to long-read high-throughput sequencing (LRS) on a MinION platform (Oxford Nanopore Technologies, Oxford, UK). The long reads generated by MinION were assembled using Canu v. 1.6 (Koren et al., 2017) and polished with the short reads generated by HiSeq using Pilon v1.22 (Walker et al., 2014) to obtain the whole genome and complete plasmid sequences. The chromosome and plasmid sequences were annotated using the prokaryotic gene prediction tool Prokka (Seemann, 2014). The plasmid type and the resistant genes were identified using the CGE server (https://cge.cbs.dtu.dk). The phylogenetic tree was constructed using the BacWGSTdb software (Institute of Translational Medicine, Zhejiang University, China) based on the previously reported single nucleotide polymorphism strategy (Ruan and Feng, 2016). For MLST, the Oxford scheme was used, and the sequence type (ST) allocation was carried out using the MLST database (https://bigsdb.pasteur.fr/klebsiella/klebsiella.html). Notably, the sequence data have been deposited in NCBI with the accession number PRJNA806839.

### Conjugation experiment

For assessing the transferability of carbapenemase-producing genes and identify whether these genes were located on plasmids, conjugation experiment was carried out as previously reported (Zeng et al., 2021). Briefly, the recipient (*E. coli* EC600) and donor strains (carbapenem-resistant *K. pneumoniae*, CRKP) were both mixed in a 4:1 ratio in Luria–Bertani broth; then, the mixtures were placed on a membrane, which was followed by incubation at 35°C for 24 h. Transconjugant selection was done on meropenem (1 μg/mL)- and rifampicin (600 μg/mL)-supplemented Mueller–Hinton agar plates. Next, the colonies that grew on the selective medium were identified using MALDI-TOF MS. Transconjugants were identified as carbapenemase-producing strains that exhibited a higher MIC of resistance to carbapenems than EC600, and then, PCR was used to confirm the presence of resistance determinants.

### Statistical analyses

In patients with cKP and hvKP strains, the following aspects were compared: clinical characteristics, biofilm formation, and antimicrobial susceptibility. For categorical variables, the Fisher’s exact test or *χ^2^* test was used, and for continuous variables, the Student’s t-test was used. For statistical analysis, SPSS 21.0 was used, and *P* < 0.05 indicated statistical significance.

## Results

### Clinical characteristics of cKP and hvKP

We isolated a total of 121 *K. pneumoniae* strains from urine samples of 121 patients with UTIs. The mean age of these patients was 57.53 years (range, 1–96 years). The demographic data of the patients revealed that most patients had underlying diseases, including bacteremia (97, 80.2%), hypertension (49, 40.5%), diabetes mellitus (37, 30.6%), and bladder catheter-related infection (35, 28.9%) (**Table 1**).

**Table 1:** Clinical characteristics and susceptibility of hvKP and cKP isolates.

	hvKP (*n* = 30, 24.7%)	Non- hvKP (*n* = 91, 75.3%)	*P* value
Age, median	59.5	56.9	0.4676
Male sex	17 (56.7%)	45 (49.5%)	0.4929
Inpatients	21 (70.0%)	81 (89.0%)	0.0283
**Underlying disease**			
Diabetes mellitus	10 (33.3%)	27 (29.7%)	0.7057
Hypertension	12 (40.0%)	37 (40.7%)	0.9491
Bacteremia	22 (73.3%)	75 (82.4%)	0.2792
Catheter	10 (33.3%)	25 (27.5%)	0.5392
Complicated UTI	23 (76.7%)	54 (59.3%)	0.0871
Biofilm forming ability	21 (70.0%)	80 (87.9%)	0.0447
C-reactive protein, mean ± SD	71.62 ± 98.58	40.10 ± 38.31	0.0640	
PCT, mean ± SD	11.06 ± 39.83	2.22 ± 9.27	0.2820	
**Antimicrobial susceptibility**			
CRE	17 (56.7%)	21 (23.1%)	0.0010
ESBLs	1 (3.3%)	35 (38.5%)	0.0003
TZP	17 (56.7%)	20 (22.0%)	0.0003
CAZ	17 (56.7%)	37 (40.7%)	0.1261
CRO	20 (66.7%)	53 (58.2%)	0.4134
FEP	18 (60.0%)	29 (31.9%)	0.0061
ATM	19 (63.3%)	37 (40.7%)	0.0308
IPM	17 (56.7%)	16 (17.6%)	0.0000
MEM	17 (56.7%)	16 (17.6%)	0.0000
AK	15 (50.0%)	12 (13.2%)	0.0000
CIP	21 (70.0%)	66 (72.5%)	0.7894
SXT	11 (36.7%)	43 (47.3%)	0.3118
F	23 (76.7%)	59 (64.8%)	0.2292
TGC	3 (10.0%)	15 (16.5%)	0.5689
PB	0 (0)	2 (2.2%)	1.0000
CZA	0 (0)	4 (4.4%)	1.0000

PCT, procalcitonin; TZP, piperacillin/tazobactam; CAZ, ceftazidime; CRO, ceftriaxone; FEP, cefepime; ATM, aztreonam; IPM, imipenem; MEM, meropenem; AK, amikacin; CIP, ciprofloxacin; SXT, trimethoprim/sulfamethoxazole; F, nitrofurantoin; TGC, tigecycline; CZA, ceftazidime/avibactam; PB, polymyxin B.

**Figure 1:**
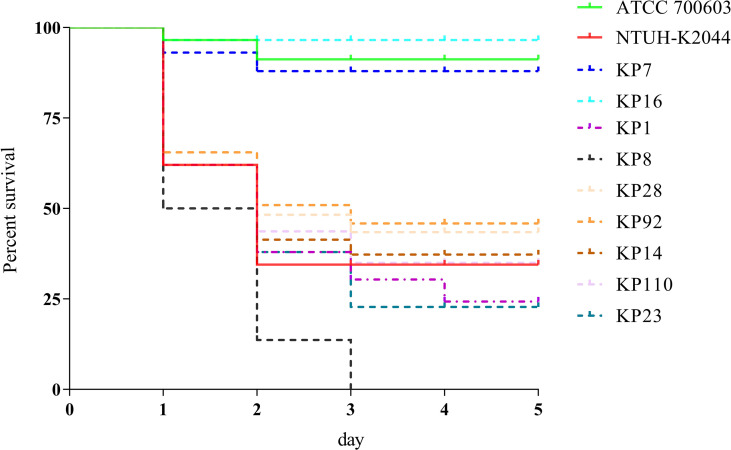
Evaluation of the virulence of KP isolates. Using a *G. mellonella* infection model, we investigated the virulence of 1 × 10^6^ CFU of each KP strain (KP1, KP7, KP8, KP14, KP16, KP23, KP28, KP92, and KP110). NTUH-K2044 and ATCC 700603 were used as high- and low-virulence KP controls, respectively. The survival rates of KP7- and KP16-infected *G. mellonella* were similar with ATCC 700603, while those of KP1, KP8, KP14, KP23, KP28, KP92, and KP110 were similar with NTUH-K2044, indicating that they were hypervirulent.

To evaluate the virulence of these strains, the virulence-associated genes were identified and confirmed using the *G. mellonella* infection model. Among the 121 *K. pneumoniae* strains, 30 (24.7%) strains simultaneously carried at least two virulence genes (*_p_rmpA*, *_p_rmpA2*, *iroB*, *peg344*, and *iucA*), and the mortality rate of *G. mellonella* infected with these 30 strains was 50%–100%, which was significantly higher than that of those infected with ATCC 700603, suggesting that these strains were hvKP (**Figure 1**).

The age, sex, underlying disease, cUTI incidence, and inflammatory indices (e.g., CRP and PCT) did not significantly differ between patients with hvKP and cKP infections. In contrast to hvKP, cKP isolates were mainly derived from inpatients (70.0% *vs.* 89.0%, *P* = 0.0283) and had superior biofilm-forming ability (70.0% *vs.* 87.9%, *P* = 0.0447). In addition, carbapenem resistance was significantly more prevalent in hvKP than in cKP (56.7% *vs.* 23.1%, *P* = 0.0010), and hvKP showed significantly more resistance than cKP to most antimicrobials, including TZP, FEP, ATM, IPM, MEM, and AK, whereas resistance to CAZ, CRO, CIP, SXT, and F did not significantly differ between the two. The detection rate of extended-spectrum β-lactamase (ESBL)-producing strains was higher in cKP than in hvKP (3.3% *vs.* 38.5%, *P* = 0.0003) (**Table 1**).

### Antimicrobial susceptibility, virulence-associated genes, and resistant genes of hvKP

**Figure 2:**
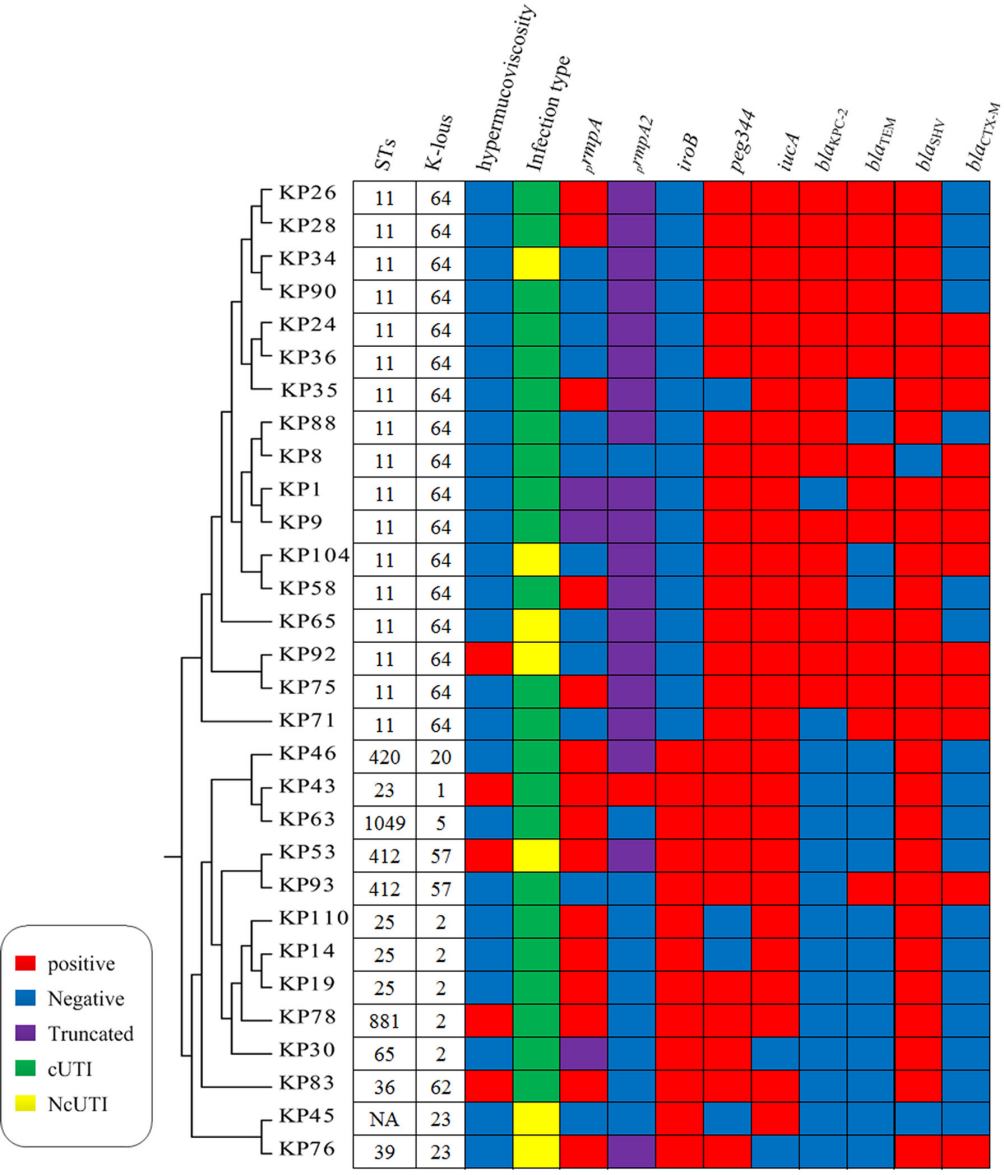
Molecular characterization of 30 hvKP isolates. NA, not determined. Homology analysis of 30 hvKP isolates was performed using BacWGSTdb software, and all K64 ST11 CR-hvKP strains belonged to the same cluster.

Among the 30 hvKP isolates, 17 were carbapenem-resistant Enterobacterales (CRE) and 1 was an ESBL-producing strain (**Table 1**). The resistance rates of hvKP to TZP, CAZ, CRO, FEP, ATM, IPM, MEM, AK, CIP, SXT, and F were 56.7%, 56.7%, 66.7%, 60.0%, 63.3%, 56.7%, 56.7%, 50.0%, 70.0%, 36.7%, and 76.7%, respectively. There were 3 TGC-resistant strains, and no isolate was resistant to polymyxins B and ceftazidime/avibactam. In addition, 15 in 17 CR-hvKP isolates carried the *bla*_KPC-2_ gene, and the remaining harbored mutations in the Ompk37-encoding gene, suggesting that the *bla*_KPC-2_ gene is the main reason for their resistance to carbapenems (**Figure 2**).

The results of the string test revealed that 13 (five hvKP and eight cKP strains) of 121 KP strains showed hypermucoviscosity. Furthermore, *_p_rmpA* and *_p_rmpA2* were identified as important regulators of the formation of the capsular polysaccharide of *K. pneumoniae*, and 18, 20 were detected in hvKP, respectively. Notably, the *_p_rmpA* gene was truncated in three hvKP strains (KP1, KP9, and KP30), and the *_p_rmpA2* gene was truncated in 19 hvKP strains (**Figure 2**).

Eight serotypes were detected, including K1, K2, K5, K20, K23, K57, K62, and K64, with the rates of 3.3% (1/30), 16.7% (5/30), 3.3% (1/30), 3.3% (1/30), 6.7% (2/30), 6.7% (2/30), 3.3% (1/30), and 56.7% (17/30), respectively. K64 was the predominant serotype in our study. In addition, these serotypes were divided into three groups (K64, K1/K2, and others), and the drug resistance and virulence of different groups of strains were compared. Herein, 16 of 17 K64 isolates were CRE and 15 carried the *bla*_KPC-2_ gene, which is predominantly responsible for conferring carbapenem resistance to *K. pneumoniae* in China. Moreover, one of six K1/K2 isolates was CRE and did not carry the *bla*_KPC-2_ gene, and seven isolates of other serotypes were not detected as CRE. K64 isolates showed 64.7% resistance to SXT, and the resistance rates of the remaining tested antimicrobials were above 82.4% and showed higher resistance rates to all tested antimicrobials than the other two groups. The distribution of the mucoid phenotype regulating genes *_p_rmpA* and *_p_rmpA2* differed among the three groups: *_p_rmpA2* was mainly distributed in the K64 group with all truncated gene sequences; *_p_rmpA* was mainly distributed in the K1/K2 group; and the other group had both distributions. The highest mortality rate in infected *G. mellonella* was observed in the K1/K2 group, followed by the other group and the K64 group (**Table 2**).

**Table 2:** Comparison of drug resistance and virulence among K64, K1/K2 and other serotype hvKP.

	K64 (*n* = 17)	K1/K2 (*n* = 6)	Others (*n* = 7)
CRE	16 (94.1%)	1 (16.7%)	0
*bla*_KPC-2_	15 (88.2%)	0	1 (14.3%)
Antimicrobial susceptibility			
TZP	16 (94.1%)	1 (16.7%)	0
CAZ	16 (94.1%)	1 (16.7%)	0
CRO	17 (100.0%)	2 (33.3%)	2 (28.6%)
FEP	17 (100.0%)	1 (16.7%)	0
ATM	17 (100.0%)	1 (16.7%)	1 (14.3%)
IPM	16 (94.1%)	1 (16.7%)	0
MEM	16 (94.1%)	1 (16.7%)	0
AK	14 (82.4%)	1 (16.7%)	0
GEN	14 (82.4%)	1 (16.7%)	0
CIP	17 (100.0%)	3 (50.0%)	1 (14.3%)
LEV	17 (100.0%)	1 (16.7%)	0
SXT	11 (64.7%)	0	0
F	17 (100.0%)	4 (66.7%)	2 (28.6%)
mortality rate of *G. mellonella*, median	56.0%	70.0%	62.0%
*_p_rmpA*	7 (41.2%)	6 (100.0%)	5 (71.4%)
*_p_rmpA2*	16 (94.1%)	1 (16.7%)	3 (42.9%)

TZP, piperacillin/tazobactam; CAZ, ceftazidime; FEP, cefepime; ATM, aztreonam; IPM, imipenem; MEM, meropenem; AK, amikacin; GEN, gentamicin; CIP, ciprofloxacin; LEV, levofloxacin; SXT, trimethoprim/sulfamethoxazole; F, nitrofurantoin.

### Molecular characteristics

We identified the following 10 ST types in the 30 hvKP isolates by MLST: ST11 (*n* = 17), ST23 (*n* = 1), ST25 (*n* = 3), ST36 (*n* = 1), ST39 (*n* = 1), ST65 (*n* = 1), ST412 (*n* = 2), ST420 (*n* = 1), ST881 (*n* = 1), and ST1049 (*n* = 1); moreover, one novel ST type was identified as well. K64 hvKP isolates were all ST11. K1 isolates were ST23, and K2 isolates were ST25, ST65, and ST881; K23 and K62 isolates were ST39 and ST36, respectively. Homology analysis of 30 hvKP isolates was performed using BacWGSTdb software (Institute of Translational Medicine, Zhejiang University, China), and all K64 ST11 CR-hvKP strains belonged to the same cluster, suggesting clonal transmission. Other serotype isolates were clustered in another clusters (**Figure 2**).

### Conjugation tests

The conjugation tests were performed on *bla*_KPC-2_-positive hvKP strains, and the results showed that two K64 ST11 CR-hvKP strains (KP88 and KP92) successfully transferred the *bla*_KPC-2_ gene into the recipient strain (EC600). In addition, transconjugants (KP88* and KP92*) were concurrently resistant to CAZ, TZP, FEP, ATM, MEM, IPM, and LEV, and KP92* also showed resistance to AK, suggesting that the genes responsible for resistance to these agents were also transferred to the recipient strain. Simultaneously, we also tested virulence genes by PCR methods in KP88* and KP92*; however, these were all negative (**Table 3**).

**Table 3:** Susceptibility of KP88, KP88*, KP92, KP92*, and EC600.

Strains	MIC (μg/mL)											
	TZP	CAZ	FEP	ATM	IPM	MEM	AK	LEV	F	CZA	TGC	PB
KP88	>512/4	64	>128	>128	>32	>32	≤4	>16	512	≤1/4	0.5	0.5
KP88*	>512/4	8	8	128	8	8	≤4	2	8	≤1/4	0.25	≤0.25
KP92	>512/4	>128	>128	>128	>32	>32	>512	>16	256	2/4	0.25	0.5
KP92*	512/4	128	32	>128	16	4	>512	2	≤4	≤1/4	≤0.125	0.5
EC600	2	≤1	≤1	≤1	≤0.25	≤0.25	≤4	≤0.125	8	≤1/4	≤0.125	0.25

KP88*, KP92*, Transconjugants; TZP, piperacillin/tazobactam; CAZ, ceftazidime; FEP, cefepime; ATM, aztreonam; IPM, imipenem; MEM, meropenem; AK, amikacin; LEV, levofloxacin; F, nitrofurantoin; CZA, ceftazidime/avibactam; TGC, tigecycline; PB, polymyxin B.

The sequence of the KP92 strain comprises a chromosome and three plasmids (plasmids 1, 2, and 3). Plasmid 1 (size, 144551 bp) is an IncFII plasmid harboring *bla*_KPC-2_, *bla*_SHV-12_, *bla*_CTX-M-65_, and *bla*_TEM-1B_ acquired antimicrobial resistance genes. Plasmid 2 (size, 216439 bp) is an IncFIB plasmid that harbors *rmpA2* (258 bp, truncated *rmpA2*), *iucABCD*, and other virulence genes. Finally, Plasmid 3 (size, 47106 bp) is an incN plasmid harboring *qnrs1* resistance gene (**Table 4**). The genetic environment of the *bla*_KPC-2_ gene is IS*26*–*tnpR*–IS*Kpn27*–*bla*_KPC−2_–IS*Kpn6*–*korC*–ORF–*klcA*–IS*Kpn19*, which is highly similar to the CP065952.1 sequence identified in patients of Taipei Veterans General Hospital.

**Table 4:** Genome characteristics of isolate KP92.

Genetic context	Length (bp)	Plasmid type	Acquired antimicrobial resistance genes	Virulence genes
Chromosome	5,392,339	–	*bla*_SHV-155_, *fosA*	*mrkABCDFHJ*, *ybtPQSX–ybtAUTE*
Plasmid 1	144,551	IncFII (pHN7A8)	*bla*_KPC-2_, *bla*_SHV-12_, *bla*_CTX-M-65_, *bla*_TEM-1B_	–
Plasmid 2	216,439	IncFIB (K) (pCAV1099-114)	–	*rmpA2*, *iucABCD*
Plasmid 3	47,106	IncN	*qnrS1*	–

-: not detected.

## Discussion

The emergence of hypervirulent *K. pneumoniae* makes clinical anti-infective therapy extremely challenging. HvKP was originally reported in patients with liver abscesses and is currently found to cause several diseases, such as pneumonia and endophthalmitis; however, it has been less frequently reported in UTIs (Russo and Marr, 2019; Zafar et al., 2019; Taraghian et al., 2020). In this study, we screened hvKP isolated from the urine samples of patients diagnosed as having UTIs and investigated the molecular and clinical characteristics of these strains to provide empirical treatment for UTIs.

The detection rate of hvKP in UTIs in our study was 24.7%, indicating a higher incidence than that reported in the study of Taraghian et al. (Taraghian et al., 2020) but lower than that reported in the study of Liu et al. (Liu et al., 2020). In addition, hvKP was predominantly derived from hospitalized patients in this study and not from community-acquired infections as in previous reports (Shankar et al., 2018; Gao et al., 2020). Diabetes has been suspected to be a risk factor for hvKP infection, but patients with hvKP and cKP did not significantly differ in this regard in this study (Zhang et al., 2016). However, this study found that compared to other serotypes, K1/K2 serotypes were associated with a higher incidence of diabetes, suggesting diabetes as a risk factor for K1/K2 hvKP infection. Consistent with our previous results, in this study as well, hvKP was found to have a weaker biofilm-forming ability than cKP (Li et al., 2022). This finding may be attributed to hvKP being more likely to escape the phagocytosis by urinary macrophages to cause bacteremia and serious consequences; the specific mechanism behind this needs to be further investigated (Kim et al., 2017). Furthermore, hvKP was more likely to cause cUTIs; however, systemic infection indicators, such as CRP and PCT, did not significantly differ between cKP and hvKP infections.

The hypervirulence combined with drug-resistant strains is associated with increasing emergence of such strains and poses a major challenge to clinical anti-infective therapy (Lee et al., 2017; Mataseje et al., 2019; Zhu et al., 2020; Zhang et al., 2021). In this study, the detection rate of CRE in hvKP was higher than it was in cKP; this could be attributed to *bla*_KPC-2_, which was identified to be successfully transferred into EC600 by conjugation test, indicating that this hypervirulent strain could acquire resistance genes by horizontal gene transfer (HGT) (Lan et al., 2021). We also confirmed the location of the *bla*_KPC-2_ gene on the IncFII plasmid, which was an important plasmid type for HGT of the *bla*_KPC-2_ gene (Andrade et al., 2011; Shi et al., 2018). The genetic environment of the *bla*_KPC-2_ gene analysis revealed that the gene was located on IS*26*–*tnpR*–IS*Kpn27*–*bla*_KPC−2_–IS*Kpn6*–*korC*–ORF–*klcA*–IS*Kpn19*, which is highly similar to the CP065952.1 sequence identified in patients of Taipei Veterans General Hospital, suggesting that the IS*26*–*tnpR*–IS*Kpn27*–*bla*_KPC−2_–IS*Kpn6* may be the core structure in the horizontal transfer of *bla*_KPC-2_. In addition, all CR-hvKP belonged to K64-ST11 and had the same cluster with the possibility of clonal transmission. ST11-KPC-2 *K pneumoniae* is the most prevalent clone in China, and it was also found in CR-hvKP (Zhang et al., 2020). Therefore, we should strengthen its monitoring and take effective control measures to prevent its further dissemination.

Initial studies suggested that hypermucoviscosity (string test > 5 mm) was an important feature of hvKP, and strains with hypermucoviscosity were considered as hvKP. However, subsequent researches have reported that the correlation between hypermucoviscosity and hypervirulence varies greatly (Fang et al., 2004; Yu et al., 2008; Lee et al., 2010; Lin et al., 2011) and that a certain proportion of cKP strains also has hypermucoviscosity (Fang et al., 2004; Yu et al., 2008; Lee et al., 2010; Lin et al., 2011), indicating that hypermucoviscosity cannot be used as a reliable method to identify hvKP. In our study, only five of 30 hvKP strains were positive for hypermucoviscocity, and eight of 91 cKP strains were also hypermucoviscous, further confirming that hypermucoviscocity was not specific to hvKP (Fang et al., 2004; Lee et al., 2010). Capsular polysaccharide is an important reason behind the pathogenicity of *K. pneumoniae*, and the pathogenic ability of different capsular polysaccharides may differ with specific serotypes. The common serotypes of hvKP are K1, K2, K5, K20, K54, and K57, mainly K1 and K2. Recently, K64 identified by *wzc* or *wzi* sequencing was considered as the main serotype of CR-hvKP, which has been seldom reported in the past due to the limitations of the traditional serotype identification methods (Pan et al., 2015). Zhang et al. (Zhang et al., 2020) reported that K64 has become a common type of CR-hvKP in China (80%, 44/55). In our study, eight serotypes, namely K1, K2, K5, K20, K23, K57, K62, and K64 (mainly K64), were detected and divided into three groups (K64, K1/K2, and others), and K64 hvKP isolates had higher resistance rate to commonly used antimicrobials but were associated with a lower mortality rate in infected *G. mellonella*, which may be attributed to the truncation of*_p_rmpA/_p_rmpA2* incurring high fitness cost in CR-hvKP (Lin et al., 2020; Shankar et al., 2021). Choi et al. (Choi and Ko, 2015) suggested that impaired virulence and reduced capsular polysaccharide production increases fitness cost in the acquisition of colistin-resistant *K. pneumoniae*. However, in the present study, the K64 serotype CR-hvKP strains were still associated with high mortality of *G. mellonella*, which is in line with the results of Shankar et al. (Shankar et al., 2021), and this may be related to the increasing simultaneous occurrence of drug-resistance and hypervirulence in *K. pneumoniae* strains observed globally. Thus, hvKP should be paid more attention to when surveillance is performed in the areas of high hvKP endemicity. The K1/K2 group had the highest mortality rate in infected *G. mellonella*, followed by the other group and the K64 group.

In summary, the clinical and molecular characteristics of hvKP isolated from UTIs were investigated in this study. Notably, compared with cKP, hvKP had higher resistance rate and was more likely to cause cUTIs. The incidence of CR-hvKP isolates was increasing among hvKP, particularly K64-ST11 CR-hvKP, which was associated with higher resistance but lower mortality in infected *G. mellonella* because of the truncation of*_p_rmpA/_p_rmpA2* incurring high fitness cost in CR-hvKP. In addition, the clonal distribution of CR-hvKP isolates was mediated by a plasmid in UTIs in our study. Therefore, surveillance of hvKP in UTIs should be strengthened.

## Data availability statement

The datasets presented in this study can be found in online repositories. The names of the repository/repositories and accession number(s) can be found below: NCBI with the accession number PRJNA806839.

## Author contributions

Study design: JL and MZ. Study conduct: FX, CM, ZL, and MT. Data collection: MT, FX, and CM. Data analysis: JL and MZ. Data interpretation: YH and HW. Drafting manuscript: JL. Revising manuscript content: MZ. Approving the final version of the manuscript: JL and MZ. All authors contributed to the article and approved the submitted version.

## Funding

The National Natural Science Foundation of China (No. 81702068), Natural Science Foundation of Hunan Province (No. 2020JJ4886, No. 2021JJ40980), the Science Foundation of Hunan Health Commission in Hunan province (No. 202111000066), and Fundamental Research Funds for the Central Universities of Central South University (No. 2021zzts1043) supported this study.

## Acknowledgments

We would like to express our gratitude to the staff of the Department of Clinical Laboratory at Central South University’s Xiangya Hospital for their assistance in collecting and identifying the *K. pneumoniae* isolates.

## Conflict of interest

The authors declare that the research was conducted in the absence of any commercial or financial relationships that could be construed as a potential conflict of interest.

## Publisher’s note

All claims expressed in this article are solely those of the authors and do not necessarily represent those of their affiliated organizations, or those of the publisher, the editors and the reviewers. Any product that may be evaluated in this article, or claim that may be made by its manufacturer, is not guaranteed or endorsed by the publisher.

## Abbreviations

hvKP, hypervirulent *K. pneumoniae;* UTI, urinary tract infection; CR-hvKP, carbapenem-resistant hypervirulent *K. pneumoniae;* cUTI, complicated urinary tract infection; KP, *K. pneumoniae;* cKP, classical *K. pneumoniae;* CFU, colony forming unit; MALDI–TOF MS, Matrix-assisted laser desorption/ionization–time-of-flight mass spectrometry; MIC, minimum inhibitory concentration; FEP, cefepime; CAZ, ceftazidime; LEV, levofloxacin; CRO, ceftriaxone; TZP, piperacillin/tazobactam; MEM, meropenem; AK, amikacin; IPM, imipenem; CIP, ciprofloxacin; GEN, gentamicin; ATM, aztreonam; SXT, trimethoprim/sulfamethoxazole; F, nitrofurantoin; CZA, ceftazidime/avibactam; TGC, tigecycline; PB, polymyxin B; PCR, polymerase chain reaction; SD, standard deviation; OD, optical density; NGS, next-generation sequencing; WGS, whole-genome sequencing; ST, sequence type; CRKP, carbapenem-resistant *K. pneumoniae;* ESBL, extended-spectrum β-lactamase; CRE, carbapenem-resistant Enterobacterales; HGT, horizontal gene transfer; PCT, procalcitonin.
